# Coordination-induced emission enhancement in gold-nanoclusters with solid-state quantum yields up to 40% for eco-friendly, low-reabsorption nano-phosphors

**DOI:** 10.1038/s41598-019-40706-3

**Published:** 2019-03-11

**Authors:** Hsiu-Ying Huang, Kun-Bin Cai, Maria Jessabel Talite, Wu-Ching Chou, Po-Wen Chen, Chi-Tsu Yuan

**Affiliations:** 1 Department of Physics, Chung Yuan Christian University, Taoyuan, Taiwan; 2 Physics Division, Institute of Nuclear Energy Research, Taoyuan, Taiwan; 3 Department of Electrophysics, National Chiao Tung University, Hsinchu, Taiwan; 4 R&D Center for Membrane Technology, Chung Yuan Christian University, Taoyuan, Taiwan

## Abstract

Colloidal quantum dots (CQDs) have gained much attention as light-emitting materials for light-conversion nano-phosphors and luminescent solar concentrators. Unfortunately, those CQDs involve toxic heavy metals and frequently need to be synthesized in the hazardous organic solvent. In addition, they suffer from severe solid-state aggregation-induced self-quenching and reabsorption losses. To address these issues, here we prepare Zn-coordinated glutathione-stabilized gold-nanocluster (Zn-GSH-AuNCs) assemblies without involving heavy metals and organic solvent. Unlike GSH-AuNCs dispersed in an aqueous solution with poor photoluminescence quantum yields (PL-QYs, typically ~1%), those Zn-GSH-AuNCs powders hold high solid-state PL-QYs up to 40 ± 5% in the aggregated state. Such Zn-induced coordination-enhanced emission (CEE) is attributed to the combined effects of suppressed non-radiative relaxation and enhanced charge-transfer interaction. In addition, they also exhibit a large Stokes shift, thus mitigating both aggregation-induced self-quenching and reabsorption losses. Motivated by these photophysical properties, we demonstrated white-light emission from all non-toxic, aqueous-synthesis nano-materials.

## Introduction

Nowadays, white-light-emitting diodes for solid-state lighting and display backlight rely on the integration of blue LEDs with rare-earth light-conversion yellow phosphors^1^. Such combination can be used to generate white light with a moderate color-rendering index and high color temperature due to the deficiency of red emission components^2^. Recently, colloidal quantum dots (CQDs) have also been applied for light-conversion nano-phosphors due to their several unique photophysical properties, such as tunable light absorption, efficient PL emission and narrow emission bandwidth, that have been commercialized in display backlight by Sony and Samsung^3^. However, those CQDs involve toxic heavy-metal elements and need to be synthesized in the hazardous organic solvent, thus would be replaced by eco-friendly, rare-earth-free nano-phosphors that can be directly synthesized in an aqueous solution^4^.

In addition to toxicity issues, CQDs also suffer from both concentration-induced PL quantum yield (PL-QY) self-quenching and reabsorption losses for serving as light-emitting materials^5–7^. When the CQDs are utilized in the solid states, the aggregation of CQDs can occur both in the thin-film or powder forms. Among CQD aggregates, the excited-state energy could be dissipated by the non-radiative relaxation through both singlet and triplet energy transfer processes, leading to concentration/aggregation induced PL-QY self-quenching^8^. The main mechanism for singlet-state quenching is dictated by dipole-mediated Forster resonance energy transfer (FRET), which strongly depends on the spectral overlap between optical absorption of the acceptors and PL emission of the donors^9^. To avoid this problem, a solid matrix, for example, organic polymer or inorganic silica needs to be introduced to disperse CQDs from the formation of aggregation^10,11^. However, the loading concentration within the organic polymer matrix by a simple physical blending method is very low due to the restriction of phase segregation and the formation of CQD aggregation, thus restricting the amount of light that can be converted^12^. Therefore, there usually exists a loading-concentration trade-off between the amount of light converted and solid-state PL-QYs for light-conversion nano-phosphor^13,14^.

In addition to concentration-induced quenching, another issue that we need to concern is the reabsorption losses for light-conversion phosphors^5–7^. When the converted light suffer from the reabsorption effect, the light intensity can be reduced accompanied with spectral red-shift, which would degrade the performance of light-conversion phosphors. Unfortunately, most of CQDs have small Stokes shift, leading to severe reabsorption losses^15,16^. To address this problem, several strategies have been applied to enlarge Stokes shift by separating the absorbing and emitting states, for example, designing heterostructured core/shell CQDs or doped CQDs^5–7^. However, both of them still rely on heavy-metal-containing CQDs or need to be synthesized in the hazardous organic solvent.

Recently, greener, aqueous-synthesis nano-materials, such as carbon nanodots (CNDs) and metal nanoclusters (NCs) have also drawn some attention in replacing those toxic, hazardous CQDs for light-conversion nano-phosphors^17–24^. Nevertheless, the CNDs also suffered from both solid-state PL-QY quenching and reabsorption losses for serving as light-conversion phosphors due to moderate Stokes shift^18,25^. In general, the PL-QY for solid-state CNDs embedded within the organic polymer matrix can be high enough only under low loading-concentration and would be significantly degraded as the concentration increases^26–28^. However, the practical performance for light-emitting phosphors depends on the overall PL emission, which is determined by both factors: the amount of light absorbed and PL-QYs. As a result, it would be beneficial for light-conversion nano-phosphors to hold high solid-state PL-QYs under high-loading concentrations.

Another newly emerging class of eco-friendly luminescent nano-materials is metal nanoclusters (NCs), including gold, copper and silver^23,29^. The AuNCs can be synthesized based on gold salt and thiolate reducing/stabilizing agents or protein template, which exhibit some promising photophysical and materials properties, such as tunable and stable PL emission, microsecond PL lifetime and good bio-compatibility^30^. Thiolate-stabilized AuNCs, such as glutathione-stabilized AuNCs (GSH-AuNCs)^31^ hold unique intra-molecular charge transfer (ICT) state, thus facilitating charge separation, which is beneficial for solar energy harvesting^32,33^. In addition, the PL emission from such AuNCs with ICT state also exhibit large Stoke shift and aggregation-induced emission enhancement (AIEE) has also motivated some promising applications, such as light-emitting materials, greener luminescent solar concentrators and bio/chemical turn-on sensing^34–37^.

Unfortunately, the main challenge for those greener NCs for “green photonics” is that the PL-QY is very poor, typically ~1% for AuNCs dispersed in an aqueous solution due to efficient non-radiative relaxation through surface-ligand motion^38^. A simple method to enhance the PL-QYs for AuNCs in solution is to induce the formation of aggregates by means of poor solvent or electrostatic attraction^39,40^. To enhance the PL-QYs in the solid state, the AuNCs can be spatially localized within the 2-D nano-sheets of layered double hydroxides, leading to high solid-state PL-QYs up to 14%, but is still less than that of conventional toxic CQDs, thus need to be further improved^41^.

In this work, to address the issues mentioned previously, Zn-coordinated glutathione-stabilized AuNCs assemblies (Zn-GSH-AuNCs) were prepared by Zn-mediated cross-linking. Interestingly, solid Zn-GSH-AuNCs powders exhibit high PL-QYs up to 40 ± 5% even in the aggregated states. Such coordination-enhanced emission (CEE) effect can be attributed to significant suppression of non-radiative relaxation and the enhancement of charge transfer interaction. The CEE effect is much better than conventional AIEE effect. In addition, their PL emission is very stable and does not suffer from concentration/aggregation induced self-quenching and reabsorption due to small spectral overlap between optical absorption and PL emission. White-light emission can be generated based on all non-toxic, aqueous-synthesis nano-materials.

## Results and Discussion

### Characterization of as-synthesized GSH-AuNCs dispersed in an aqueous solution

**Figure 1:**
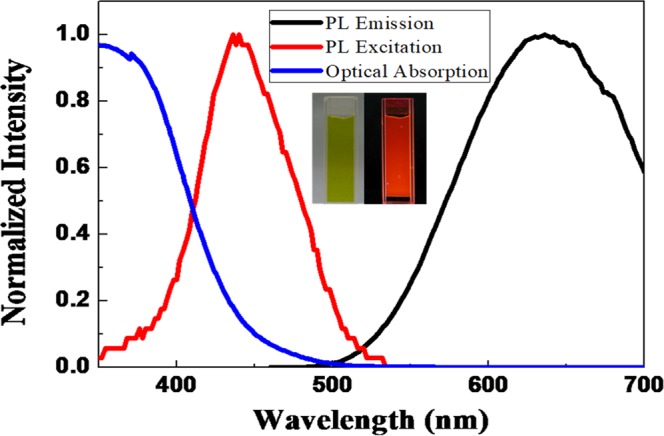
Normalized Optical absorption, PL emission and PL excitation spectra for GSH-AuNCs dispersed in an aqueous solution and the corresponding photographs under room-light and UV-light illumination.

Figure 1 shows the normalized optical absorption, PL emission and PL excitation spectra for pristine GSH-AuNCs dispersed in an aqueous solution, as well as the photographs under room-light and UV-light illumination in the inset. The optical absorption starts at ~530 nm and PL emission peak is centered at ~640 nm with a small spectral overlap, which is a key photophysical property in mitigating both PL-QY self-quenching and reabsoprtion losses. The PL emission exhibits a large Stokes shift and unstructured spectral shape, which has been assigned to the transition from the ICT states^42^. In addition, the PL-excitation peak is centered at ~460 nm, which is just located at the emission peak of blue light-emitting diodes (LEDs), thus can efficiently perform light-conversion processes under blue-LED excitation. Unfortunately, the PL-QYs for as-synthesized GSH-AuNCs dispersed in an aqueous solution is only 1 ± 0.5% due to efficient non-radiative relaxation via ligand motions^32^.

**Figure 2:**
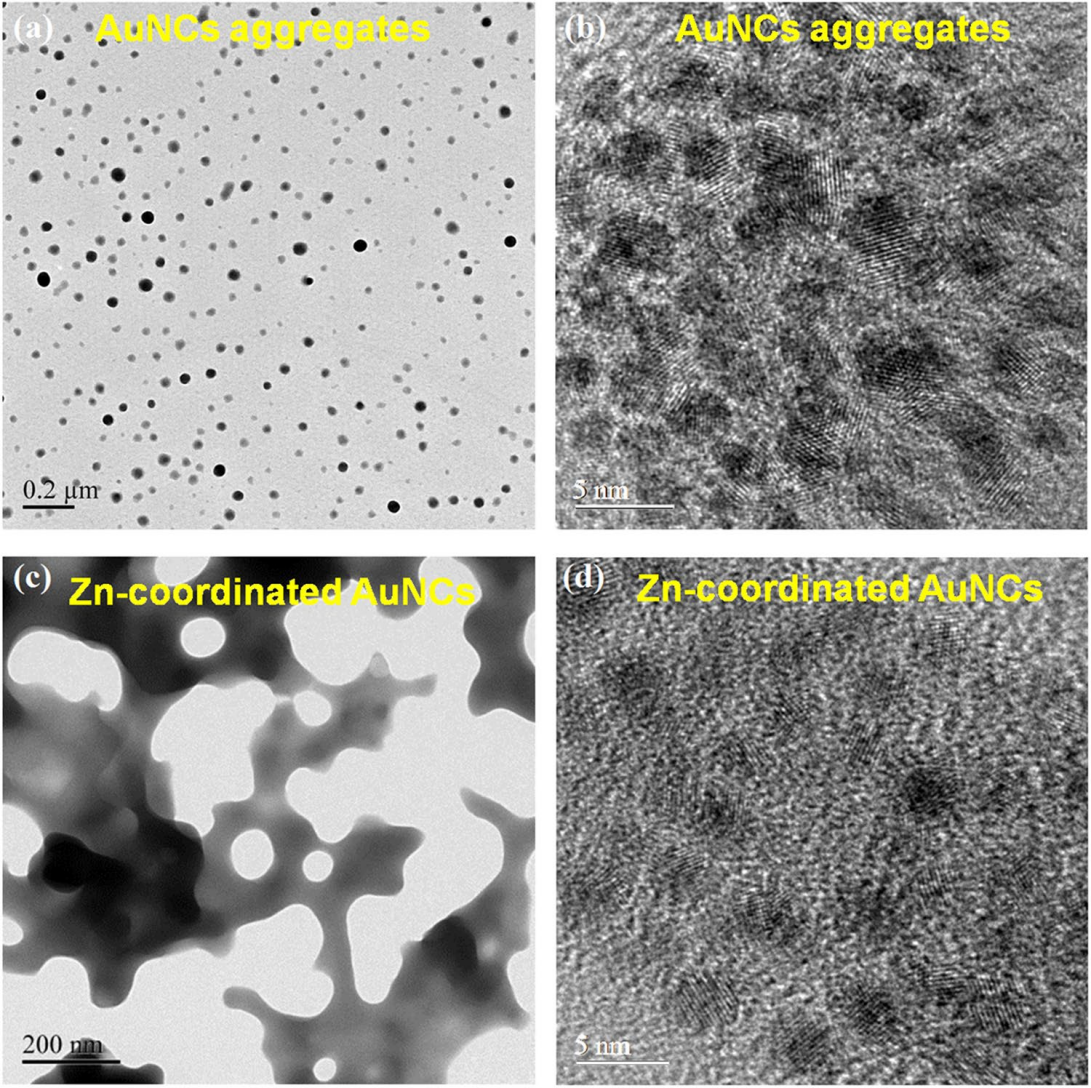
TEM images for GSH-AuNCs powders (**a**) lower magnification, (**b**) higher magnification) and GSH-AuNCs powders in the presence of Zn^2+^ (**c**) lower magnification, (**d**) higher magnification.

To enhance the PL-QYs of GSH-AuNCs, a facile method based on AIEE effect was first employed by preparing solid GSH-AuNCs aggregates using poor solvent, which can serve as the control samples for comparing with Zn-coordinated GSH-AuNCs assemblies. As shown in Fig. 2a, the GSH-AuNCs were aggregated to form spherical morphology with different sizes by the poor solvent. Such solid GSH-AuNCs aggregates with AIEE effect exhibit a moderate enhancement of solid-state PL-QYs to 5~10% due to the suppression of non-radiative decay pathways via the restriction of surface-ligand motions^39^. The PL spectrum was also recorded for the aggregates, as shown in the Supporting Information Fig. S1, which displays a spectral blue-shift as compared with pristine GSH-AuNCs dispersed in an aqueous solution. This spectral blue shift has been assigned to the changes of inter- and intra-NC Au(I)…Au(I) aurophilic interaction^39^ upon the formation of the aggregates. However, the achieved PL-QY of GSH-AuNCs by AIEE effect is still far behind that of CdSe based CQDs.

### Formation of Zn-coordinated GSH-AuNCs assemblies

To further enhance solid-state PL-QYs of GSH-AuNCs, Zn-coordination-induced assembly was employed to cross-link the GSH-AuNCs. The carboxylate functional groups on GSH ligands can be coordinated with Zn cations, leading to the formation of Zn-induced cross-linked GSH-AuNCs assemblies (hereafter abbreviated by Zn-GSH-AuNCs), as evidenced by the TEM imaging in Fig. 2c and X-ray photoelectron spectroscopy (discussed later). In contrast to spherical morphology of GSH-AuNCs (Fig. 2a) with simple AIEE effect, the Zn-GSH-AuNCs assemblies display irregular network morphology and is highly stable even under ambient environment for several months (Fig. 2c). From high-resolution TEM images shown in Fig. 2(b–d), clear fringe patterns were found, indicating high crystallinity of both samples and the average sizes are ~2.1 ± 0.2 nm for individual AuNCs.

**Figure 3:**
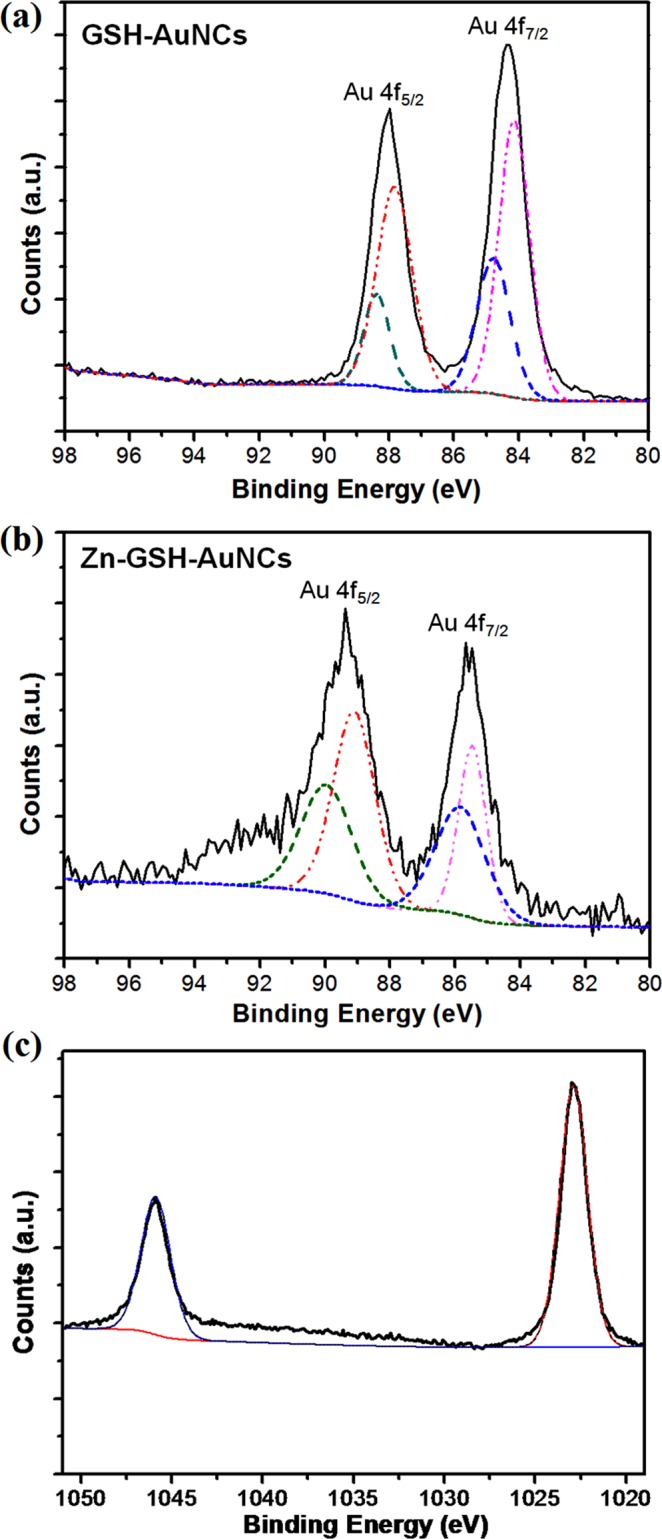
(**a**,**b**) XPS spectra for GSH-AuNCs and Zn-GSH-AuNCs within the range of Au 4f binding energy and (**c**) XPS spectrum for Zn-GSH-AuNCs within the range of Zn binding energy.

To further unravel the modification of GSH-AuNCs upon Zn-induced coordination, X-ray photoelectron spectroscopy (XPS) measurements were performed for both GSH-AuNCs and assembled Zn-GSH-AuNCs. The XPS is a useful technique that can be employed to investigate the valence states of AuNCs^39^. It has been proposed that the ratio of the integrated area of Au(I) and Au(0) species in XPS plays a critical role in determining the PL-QYs^43^. For example, the PL-QYs can be enhanced from ~1% to ~10% by the sulfur oxidation of AuNCs^44^. As shown in Fig. 3(a,b), the XPS curves within Au 4f binding energy range for both samples show typical two bumps, corresponding to the binding energy of 4f_7/2_ and 4f_5/2_ contributions (black lines). Each 4f bump can be further deconvoluted by two components, corresponding to Au(0) (lower binding energy) and Au(I) (higher binding energy) contributions (for Au 4f_5/2_ peaks, red line is A(0) component and green line is Au(I) component)^39^. Clearly, both 4f peaks of Zn-GSH-AuNCs are shifted to higher binding energy as compared with that of GSH-AuNCs, implying enhanced charge transfer interaction. Previous report has also shown that the enhanced charge transfer interaction between the metal cores and surface ligands can increase the binding energy of Au cores^45^. For Zn-GSH-AuNCs, the ratio of integrated areas between Au(I) and Au(0) species was increased and an additional peak corresponding to Zn contribution appeared shown in Fig. 3(c), indicating the formation of Zn-GSH-AuNCs assemblies.

### High solid-state PL-QYs in Zn-GSH-AuNCs assemblies

**Figure 4:**
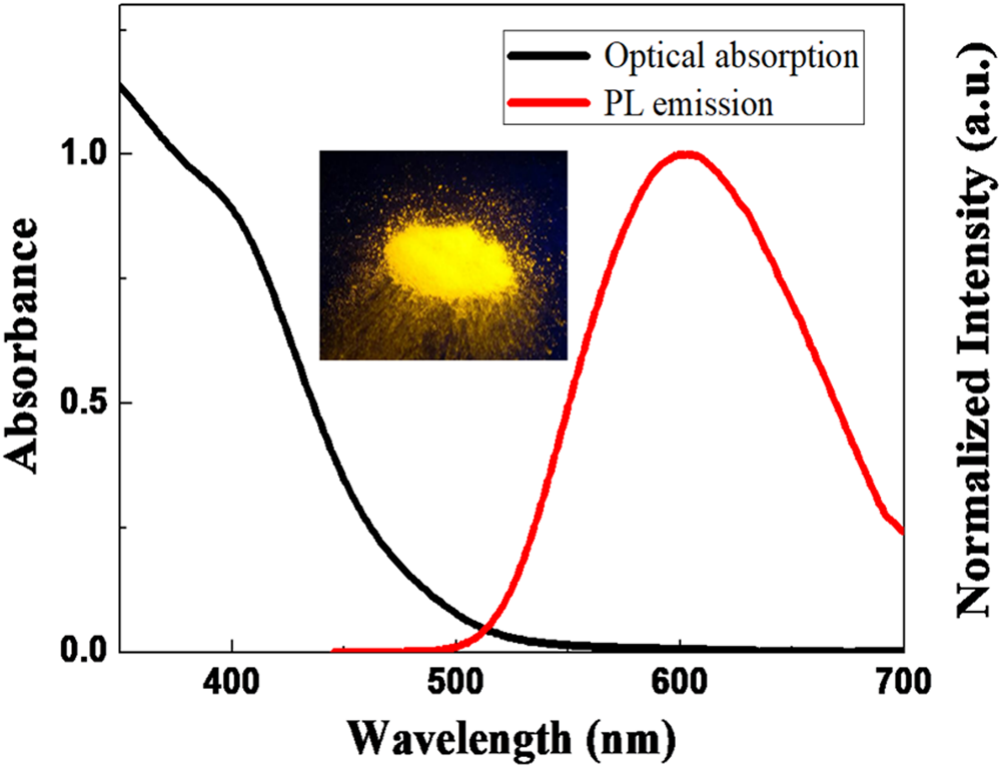
Optical absorption and normalized PL emission for Zn-GSH-AuNCs powders and the corresponding photograph under UV illumination.

In addition to the variations in morphology and valence states, the photophysical properties were also significantly modified for Zn-GSH-AuNCs assemblies. As shown in Fig. 4, solid Zn-GSH-AuNCs powders exhibit bright PL emission centered at ~602 nm, which is similar to solid GSH-AuNCs aggregates but is largely blue-shifted as compared with pristine GSH-AuNCs dispersed in solution. In light of high-resolution TEM imaging shown in Fig. 2d, we did not see obvious size variations for individual NCs within the assemblies, thus ruling out the size effect on the observed spectral blue-shifting. In addition, aggregation-induced non-radiative energy transfer should result in spectral red-shifting of PL emission accompanied with PL quenching, which are in contrast to our experimental findings, thus this can be also excluded. Such large PL blue-shift can be again attributed to assembly-induced alteration of Au(I)…Au(I) aurophilic interaction^46^. After the formation of Zn-GSH-AuNCs assemblies, inter-AuNCs aurophilic interaction was enhanced, while intra-AuNCs interaction was reduced accordingly, leading to spectral blue-shift^47^.

**Figure 5:**
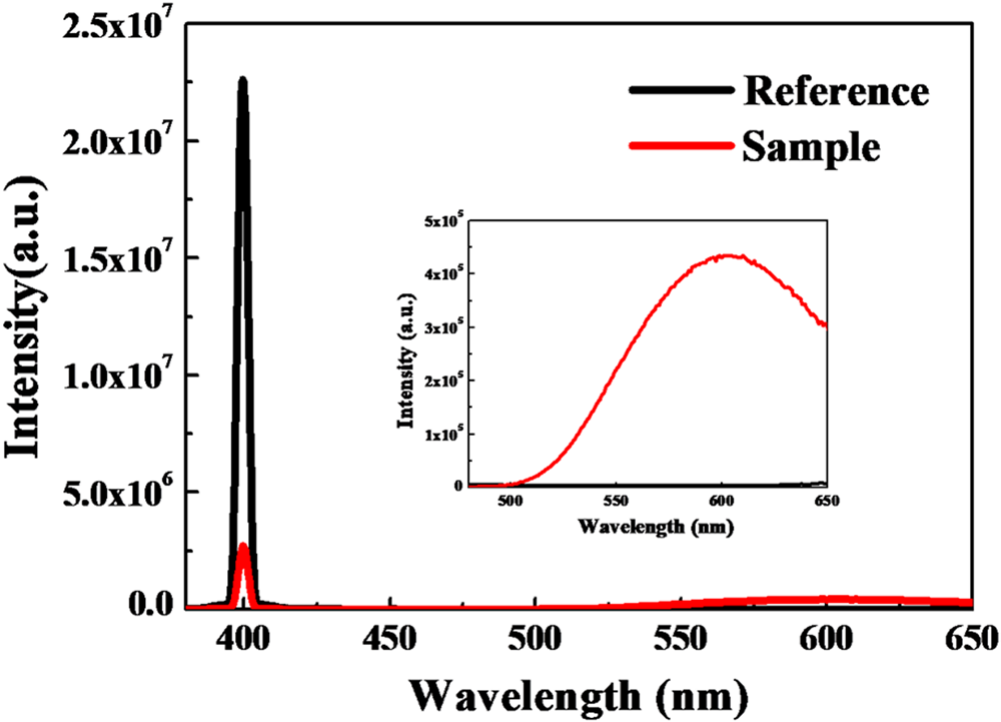
Experimental data of absolute PL-QY measurement for Zn-GSH-AuNCs.

In addition, solid-state PL-QYs of assembled Zn-GSH-AuNCs can be significantly enhanced up to 40 ± 5% even in the aggregated state, as shown in Fig. 5. The absolute PL-QY measurement was performed based on a spectrometer combined with an integrating sphere. Full-range spectra (including both absorption and emission ranges) for both experimental samples (red line) and blank reference (black line) were measured. The PL-QY value can be obtained by this equation, ${\eta }_{PLQY}=\frac{P{L}_{sample}}{{E}_{reference}-{E}_{sample}}$, where ${E}_{reference},{E}_{sample}$ represent the intensity of excitation light not absorbed by the reference samples and experimental samples, while $P{L}_{sample}$ denotes the PL intensity of the experimental samples. We found that the CEE effect on the PL-QY enhancement is larger than that of conventional AIEE method.

**Figure 6:**
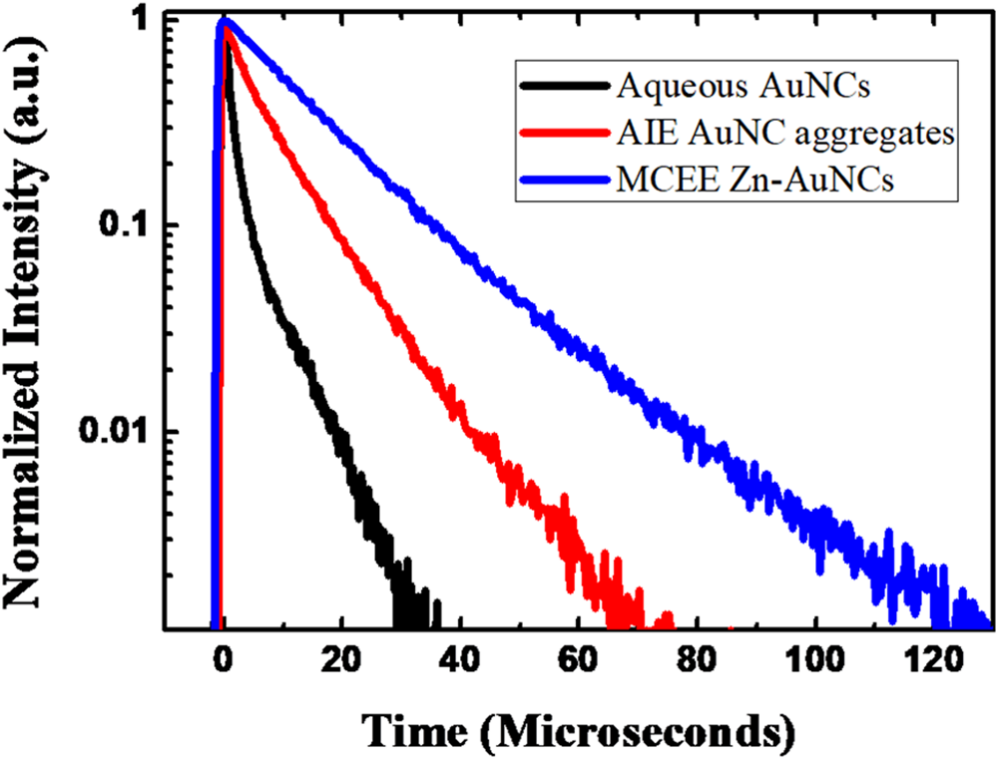
Time-resolved PL decay curves for GSH-AuNCs dispersed in solution, solid GSH-AuNCs powders and Zn-GSH-AuNCs powders.

To further unravel large PL-QY enhancement due to the CEE effect, time-resolved PL decay curves were measured for AuNCs dispersed in solution, AuNCs powders with AIEE and Zn-GSH-AuNCs with CEE effects, as shown in Fig. 6. We found that the PL lifetimes can be clearly lengthened after the formation of physical aggregates of GSH-AuNCs, and even more longer for Zn-GSH-AuNCs assemblies with CEE effect. To quantitatively analyze the PL decay profiles, stretched exponential functions, $I(t)={I}_{0}\,\exp {(-\frac{t}{\tau })}^{\beta }+{I}_{b}$ were employed to fit the experimental data, where ${I}_{0},\beta ,\tau ,{I}_{b}$ represent the PL intensity at zero time delay, stretching parameter, characteristic time, and background intensity, respectively (more detailed fitting results can be found in supplementary data). The average PL lifetimes can be calculated using this equation, $\langle \tau \rangle =\frac{\tau }{\beta }{\rm{\Gamma }}(\frac{1}{\beta })$, where ${\rm{\Gamma }}$ is the gamma function^48^, which are ~1.2 *μs*, ~4.4 *μs*, and ~10.3 *μs* for GSH-AuNCs dispersed in an aqueous solution, aggregated GSH-AuNCs and assembled Zn-GSH-AuNCs (Supporting Information Fig. S2). It is known that the PL-QY is defined by this equation, ${\eta }_{QY}=\frac{{k}_{r}}{{k}_{r}+{k}_{nr}}={\tau }_{PL}\times {k}_{r}$, where ${k}_{r},{k}_{nr},{\tau }_{PL}$ represent the radiative decay rates, non-radiative decay rates, and PL lifetimes, respectively. It implies that the non-radiative relaxation can be significantly reduced by the CEE effect, leading to enhanced PL-QYs and lengthened PL lifetime. In addition, such CEE-induced PL enhancement is much better compared with that of conventional AIEE effect.

In general, the PL-QY enhancement by physical aggregations can be attributed to the restriction of surface-ligand vibration and rotation, thus suppressing non-radiative relaxation^39^. In this case, the enhanced PL-QYs along with suppressed non-radiative decay rates should be observed without changing other photophysical and chemical properties, such as spectral properties, radiative decay processes and oxidation states, which are in stark contrast to the data we observed here. However, the AIEE effect in metal NCs would be more complex due to the introduction of extra metal-metal and metal-ligand interactions. The PL emission mechanism for GSH-AuNCs can be attributed to the ligand-to-metal intra-molecular charge transfer state, thus would be modified by extra metallophilic interaction and metal-ligand interaction. Upon the formation of AuNCs aggregates or assemblies, the intra- and inter-NC metallophilic interaction could be also altered, thus changing PL spectral properties and QYs by the variation of intra-molecular charge transfer interaction.

By comparing dispersed GSH-AuNCs solution with aggregated GSH-AuNCs powders, in addition to PL-QY enhancement, the PL emission was spectrally blue-shifted, implying the modification of metallophilic interaction. An important finding is that the assembled Zn-GSH-AuNCs exhibit unprecedented high solid-state PL-QYs up to 40 ± 5%, which is larger than that of aggregated GSH-AuNCs with AIEE effect. In light of our experimental data, we can deduce the radiative and non-radiative decay rates using this simple equation, ${\eta }_{QY}=\frac{{k}_{r}}{{k}_{r}+{k}_{nr}}={\tau }_{PL}\times {k}_{r}$, where ${k}_{r},{k}_{nr},{\tau }_{PL}$ denote radiative decay rates, non-radiative decay rates, and average PL lifetime, respectively. The ${k}_{r},{k}_{nr}$ values are $8.3\times {10}^{3}{s}^{-1}$, $8.3\times {10}^{5}{s}^{-1}$ for dispersed GSH-AuNCs; $1.8\times {10}^{4}{s}^{-1}$, $2.1\times {10}^{5}{s}^{-1}$ for aggregated GSH-AuNCs; $3.9\times {10}^{4}{s}^{-1}$ and $5.8\times {10}^{4}{s}^{-1}$ for assembled Zn-GSH-AuNCs. Interestingly, the radiative decay rates can be enhanced while the non-radiative decay rates can be suppressed for assembled Zn-GSH-AuNCs compared with both GSH-AuNCs samples.

### Characterization of reabsorption effect in Zn-GSH-AuNCs

**Figure 7:**
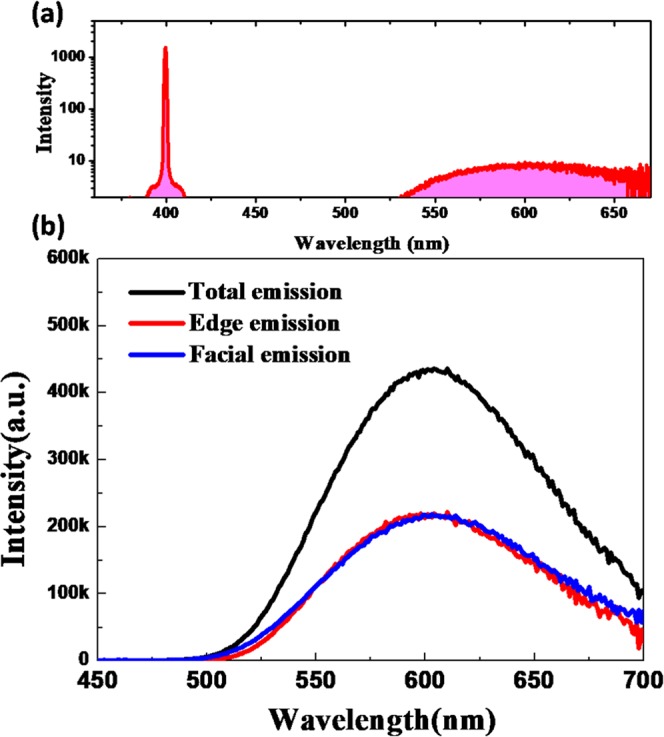
(**a**) Absolute PL-QY measurement for Zn-GSH-AuNCs embedded in PVB matrix and (**b**) PL emission spectra collected from face and edge sides of the luminescent slab.

It is known that multiple reabsorption events would significantly reduce the PL intensity and spectral properties, thus largely degrading the performance of light-conversion materials^5–7^. To characterize the reabsorption effect, the Zn-GSH-AuNCs were mixed with a polymer host to form a luminescent slab. Interestingly, the PL-QY of the luminescent slab is even higher up to ~43%, as shown in Fig. 7a, which might be due to the protection of local environment of Zn-GSH-AuNCs assemblies by the rigid polymer matrix. Such a luminescent slab can serve as a waveguide to trap a fraction of PL emission by total internal reflection towards the edge of the slab; while a fraction of emitted light can be directly radiated out of the slab through the escape cone. In this case, the edge emission would undergo more reabsorption processes. As a result, by comparing the PL emission collected either at the edge or from the face for the luminescent slab, the reabsorption effect can be assessed, which has been widely adopted to evaluate the reabsorption losses in luminescent solar concentrators^35^. As shown in Fig. 7b, the PL emission collected from the surfaces and the edges are nearly identical except for a slight reduction in the high-energy side, implying very small reabsorption effects, which can be expected due to small spectral overlap between optical absorption and PL emission spectra.

### Resistance to aggregation-induced PL-QY self-quenching

In general, the PL-QYs of colloidal nano-materials, such as colloidal QDs and organic dyes could be significantly reduced in the solid state due to the formation of the aggregates, in which, non-radiative FRET processes could be introduced^8,9^. Fortunately, such negative effects play a minor role in Zn-GSH-AuNCs due to unique small spectral overlap integral. The FRET efficiency can be expressed by this equation, ${E}_{FRET}=\frac{1}{1+{(d/{R}_{0})}^{6}}$, where $d,{R}_{0}$ denote the separation distances between the samples and Forster radius^9^. The Forster radius can be calculated by this formula, ${R}_{0}=0.211{[{\kappa }^{2}{n}^{-4}{\varphi }_{D}J(\lambda )]}^{1/6}$, where $\kappa ,n,{\varphi }_{D},J(\lambda )$ represent orientation factor, refractive index, quantum yields and spectral overlap integral, respectively. As a result, the Foster radius strongly depends on the spectral overlap integral, defined as $J(\lambda )={\int }_{0}^{\infty }{F}_{D}(\lambda ){\varepsilon }_{A}(\lambda ){\lambda }^{4}d\lambda$, where ${F}_{D}(\lambda ),\varepsilon (A)$ is the normalized PL spectrum of the donors and molar extinction coefficient of the acceptors. The derived Forster radius of Zn-GSH-AuNCs is around 0.9~1.0 nm (Supplementary Information Fig. S3). A plot of FRET efficiency as a function of the separation distance is displayed in the Supporting Information Fig. S3 based on the derived Forster radius. From TEM imaging, we found that most of Zn-GSH-AuNCs do not entangle with each other, thus the mutual average distance for individual NCs within the assemblies is at least ~3 nm by considering the length of surface ligands and the radius of NCs, thus reducing the FRET efficiency.

### Resistance to photo-bleaching

**Figure 8:**
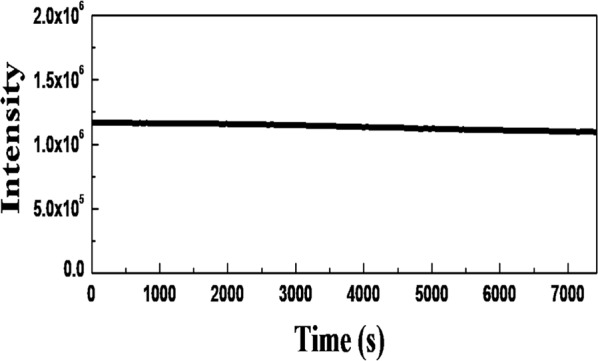
PL intensity as a function of observation time for Zn-GSH-AuNCs embedded in polymer host.

For conventional triplet-state-relevant emission, including phosphorescence and thermally activated delayed fluorescence (TADF), such triplet-related emissions with ~microseconds or longer PL lifetimes can be only observed under deaerated condition and would be significantly quenched or totally disappeared at ambient environment due to efficient triplet energy transfer to either surrounding molecular oxygen or host matrices^49–51^. In contrast, as already shown in Fig. 6, the PL decay profile with a long microsecond lifetime can be maintained under ambient environment for the Zn-GSH-AuNCs, indicating stable PL emission against triplet quenching. On the other hand, the PL intensity under UV illumination would be gradually degraded with time for organic compounds or inorganic colloidal QDs, referred to as photo-bleaching^52^. Some researchers have proposed that the triplet energy transfer plays an important role in photo-bleaching behavior^53^. Once the excited triplet energy is transferred to surrounding molecular oxygen, single oxygen and some reactive oxygen species can be generated, thus oxidizing the emitters, leading to the photo-bleaching effect. Figure 8 shows a plot of the PL intensity as a function of observation time under UV illumination. Clearly, the PL intensity is very stable under ambient environments without noticeable PL reduction. It implies that the triplet states can be stabilized free from triplet energy transfer, leading to stable PL emission.

### White-light generation based on all eco-friendly, aqueous-synthesis nano-phosphors

**Figure 9:**
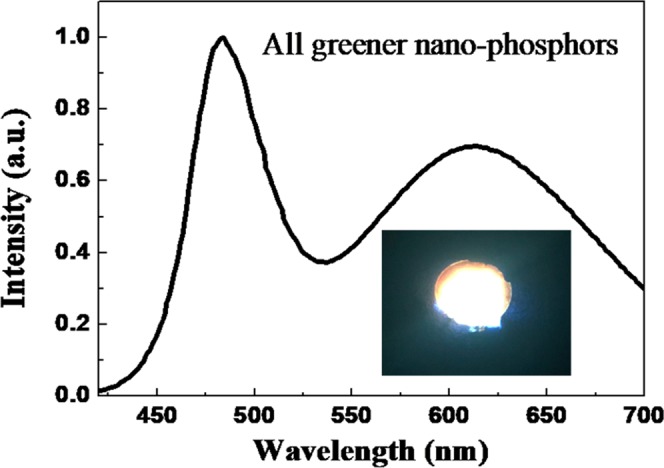
White-light generation based on all greener, aqueous-synthesis nano-phosphors integrated with blue LEDs and the corresponding photograph.

Recently, more attention has been paid on the generation of white-light emission based on greener, aqueous-synthesis, biocompatible phosphors, which are promising alternatives for commonly used rare-earth-containing micro-phosphors and heavy-metal-containing nano-phosphors^17,54–56^. The photometric properties of heavy-metal-containing nano-phosphors can be simply modified, for example by changing the sizes or compositions, thus are beneficial in improving color-rendering index and color-correlated temperature. Unfortunately, as mentioned previously, those nano-phosphors involve heavy metals and hazardous solvent and still suffer from aggregation-induced self-quenching and reabsorption losses. As a result, it is desirable to generate white-light emission based on all greener, aqueous-synthesis, rare-earth-free nano-phosphors. To this end, green-emissive carbon nano-dots and yellow-orange emissive Zn-GSH-AuNCs powders were physically blended to form multi-color nano-phosphors. By combining blue-emissive LEDs, white-light emission with a CIE index of (0.38, 0.38) and CRI value of 75 can be generated, as shown in Fig. 9 and the corresponding photograph in the inset.

### Assembly-induced emission enhancement in metal nanoclusters

Recently, assembly-induced emission enhancement or bonding-induced emission enhancement have been utilized to enhance the PL-QY of metal NCs^57,58^. For example, the PL emission from AuNCs ([Au_8_]^4+^) can be switched from fluorescence to phosphorescence by solvent-induced assembly with assembly-enhanced emission up to 18% in the solid state^59^. In addition, the assembly of AuNCs to the nano-ribbon form at the interface between oil and water can enhance the PL-QYs up to 13.3%^60^. Unfortunately, so far, the reached PL-QYs “in the solid state” for assembled AuNCs is still less than 20%, which are still far behind that of conventional colloidal toxic QDs. Our demonstration provides a facile, post-treatment method to fabricate “greener” Zn-GSH-AuNCs assemblies with a high solid-state PL-QY up to 40 ± 5% even in the aggregated state, which are even comparable to toxic colloidal QDs and concurrently possess a large Stoke shift, which would be beneficial for light-conversion nano-phosphors and other promising photonic applications. Such Zn-induced coordination-enhanced emission strategy can be also employed for GSH-stabilized copper nanoclusters (Supporting Information Fig. S4).

## Conclusion

In conclusion, Zn-coordinated GSH-AuNCs assemblies were prepared by a simple post-treatment method. Such Zn-GSH-AuNCs exhibit high solid-state PL-QYs of 40 ± 5% even in the aggregated states. This effect can be attributed to significant suppression of non-radiative relaxation and the enhancement of charge-transfer interaction, which is evidenced by time-resolved PL and XPS measurements. In addition, the PL emission is very stable under ambient condition and does not suffer from both aggregation-induced self-quenching and reabsorption losses due to large Stokes shift. Motivated by those photophysical properties, we have demonstrated white light emission based on all nontoxic, aqueous-synthesis nano-phosphors.

## Experimental Section

### Materials

Gold(III) chloride trihydrate (HAuCl_4_∙3H_2_O), L-Glutathione in the reduced form (GSH), zinc chloride, polyvinyl butyral (PVB), hydrochloric acid (HCl, 37%), sodium hydroxide and absolute ethanol were purchased from Sigma-Aldrich. All of these reagents are of analytical grade and used without further treatment.

### Synthesis of GSH-AuNCs

GSH stabilized AuNCs were prepared as follows^31,61^. Freshly prepared aqueous solution of GSH (10 mL, 75 mM) was mixed with HAuCl_4_ (10 mL, 50 mM) under vigorous stir for 5 min at ambient temperature. Until the solution turned colorless, the solution was sealed and transferred into the microwave vessels. The reaction temperature was set at 90 °C, which can be reached in 3 min from room temperature, and the irradiation time was set for 3 h (Anton Paar). Then, the pH value of GSH-AuNCs solution was adjusted to 6.0 using NaOH. The obtained product was stored in a refrigerator at 4 °C for further use. Powdered sample of GSH-AuNCs was precipitated from the supernatant by the addition of ethanol and washed with ethanol repeatedly for three times. The product was freeze-dried and then dispersed in deionized water (200 mg ml^−1^) and kept under ambient conditions before use.

### Fabrication of solid-state Zn-GSH-AuNCs assemblies

Zn-modified AuNCs solution was prepared by mixing 2 ml of ZnCl_2_ (50 mM), and 2 ml of as-prepared GSH-AuNCs solution. The pH value of those mixtures was adjusted to 6.0 using NaOH. The obtained products were stored in a refrigerator at 4 °C for further use. Powdered sample of Zn-AuNCs assemblies were precipitated from the supernatant by the addition of ethanol and washed with ethanol repeatedly for three times.

### Fabrication of luminescent Zn-GSH-AuNCs slab

In a typical synthesis, 0.3 g PVB was added into 2 mL of ethanol, sonicated for 30 min. 0.05 g of as-prepared Zn-AuNCs powders were mixed with the PVB solution and then vortexed for 30 min at room temperature. The resulting mixtures were coated on the glass and allowed to dry for 12 h at 50 °C under vacuum.

### Preparation of carbon nanodots

Carbon nanodots were prepared according to the reference^10^. The precursor solution for CD synthesis was 1 g citric acid and 2 g urea dissolved in 20 ml of de-ionized water. The precursor solution was heated by a 700 W domestic microwave oven for 3.5 minutes. The solution changed from transparent to dark-brown clustered solids. Those solids were dissolved in 40 ml of de-ionized water and centrifuged to remove the large agglomerated particles at 6200 rpm for 20 minutes to obtain carbon nanodots.

### Characterization

Transmission electron microscopy (TEM) was performed on JEOL JEM-2010 high resolution transmission electron microscope operated at 200 kV. X-ray photoelectron spectroscopy (XPS) was performed on an Thermo Fisher Scientific K-Alpha X-ray photoelectron spectrometer. FTIR spectrum was collected at room temperature (on a Jasco FTIR-4100 spectrometer) from sample prepared as pellets with KBr. The UV–Vis absorption spectrum was recorded with V-750 UV–Vis spectrophotometer (Jasco). Steady-state and time-resolved PL measurements were performed based on a spectrophotometers (Fluotime 300, PicoQuant). A pulsed Xenon lamp was used as an excitation source and the PL emission excited at ~400 nm is collected by a PMT detector with the calibration according to the wavelength-response function of our detector. The instrument response function for our whole time-resolved PL measurement system is ~400 ns. Absolute PL-QY measurement was performed based on the aforementioned spectrometer incorporated with an integrating sphere. The excitation and emission spectra were measured for both reference and experimental samples by the calibrated detectors. In this case, the ratio between total amounts of photons emitted and absorbed can be determined, thus the PL-QY can be deduced.

## Supplementary information

Supporting Information

## References

[1] Abe, S., Joos, J. J., Martin, L. I. D. J., Hens, Z. & Smet, P. F. Hybrid Remote Quantum Dot/Powder Phosphor Designs for Display Backlights. *Light: Science &Amp; Applications* **6**, e16271, 10.1038/lsa.2016.271, https://www.nature.com/articles/lsa2016271#supplementary-information (2017). 10.1038/lsa.2016.271 PMC6062237 30167259

[2] Schubert EF, Kim JK (2005). Solid-State Light Sources Getting Smart. Science.

[3] Bourzac K (2013). Quantum Dots Go on Display: Adoption by TV Makers Could Expand the Market for Light-Emitting Nanocrystals. Nature.

[4] Tian Z (2017). Full-Color Inorganic Carbon Dot Phosphors for White-Light-Emitting Diodes. Advanced Optical Materials.

[5] Kundu J, Ghosh Y, Dennis AM, Htoon H, Hollingsworth JA (2012). Giant Nanocrystal Quantum Dots: Stable Down-Conversion Phosphors that Exploit a Large Stokes Shift and Efficient Shell-to-Core Energy Relaxation. Nano Letters.

[6] Dupont D, Tessier MD, Smet PF, Hens Z (2017). Indium Phosphide-Based Quantum Dots with Shell-Enhanced Absorption for Luminescent Down-Conversion. Advanced Materials.

[7] Wang X, Yan X, Li W, Sun K (2012). Doped Quantum Dots for White-Light-Emitting Diodes Without Reabsorption of Multiphase Phosphors. Advanced Materials.

[8] Kholmicheva N, Moroz P, Eckard H, Jensen G, Zamkov M (2017). Energy Transfer in Quantum Dot Solids. ACS Energy Letters.

[9] Kholmicheva N (2017). Enhanced Emission of Nanocrystal Solids Featuring Slowly Diffusive Excitons. The Journal of Physical Chemistry C.

[10] Jiang ZC (2016). A Facile and Low-Cost Method to Enhance the Internal Quantum Yield and External Light-Extraction Efficiency for Flexible Light-Emitting Carbon-Dot Films. Scientific Reports.

[11] Wang J, Zhang F, Wang Y, Yang Y, Liu X (2018). Efficient Resistance Against Solid-State Quenching of Carbon Dots Towards White Light Emitting Diodes by Physical Embedding into Silica. Carbon.

[12] Li S (2007). Bulk Synthesis of Transparent and Homogeneous Polymeric Hybrid Materials with ZnO Quantum Dots and PMMA. Advanced Materials.

[13] Kovalchuk A, Huang K, Xiang C, Martí AA, Tour JM (2015). Luminescent Polymer Composite Films Containing Coal-Derived Graphene Quantum Dots. ACS Applied Materials & Interfaces.

[14] Lee, J., Sundar, V. C., Heine, J. R., Bawendi, M. G. & Jensen, K. F. Full Color Emission from II–VI Semiconductor Quantum Dot–Polymer Composites. *Advanced Materials* **12**, 1102–1105, 10.1002/1521-4095(200008)12:15<1102::AID-ADMA1102>3.0.CO;2-J (2000).

[15] Hanson CJ (2015). Matching Solid-State to Solution-Phase Photoluminescence for Near-Unity Down-Conversion Efficiency Using Giant Quantum Dots. ACS Applied Materials & Interfaces.

[16] Zhao B (2014). Intrinsic quantum dot based white-light-emitting diodes with a layered coating structure for reduced reabsorption of multiphase phosphors. RSC Advances.

[17] Wang X-F (2018). Towards high-powered remote WLED based on flexible white-luminescent polymer composite films containing S, N co-doped graphene quantum dots. Chemical Engineering Journal.

[18] Kwon W (2013). Freestanding Luminescent Films of Nitrogen-Rich Carbon Nanodots toward Large-Scale Phosphor-Based White-Light-Emitting Devices. Chemistry of Materials.

[19] Baekelant W, Coutino-Gonzalez E, Steele JA, Roeffaers MBJ (2017). & Hofkens, J. Form Follows Function: Warming White LEDs Using Metal Cluster-Loaded Zeolites as Phosphors. ACS Energy Letters.

[20] Coutiño-Gonzalez E (2017). Silver Clusters in Zeolites: From Self-Assembly to Ground-Breaking Luminescent Properties. Accounts of Chemical Research.

[21] Kennes K (2017). Silver Zeolite Composites-Based LEDs: A Novel Solid-State Lighting Approach. Advanced Functional Materials.

[22] Wen X, Yu P, Toh Y-R, Tang J (2012). Structure-Correlated Dual Fluorescent Bands in BSA-Protected Au25 Nanoclusters. The Journal of Physical Chemistry C.

[23] Jin R, Zeng C, Zhou M, Chen Y (2016). Atomically Precise Colloidal Metal Nanoclusters and Nanoparticles: Fundamentals and Opportunities. Chemical Reviews.

[24] Yao Q, Yuan X, Chen T, Leong DT, Xie J (2018). Engineering Functional Metal Materials at the Atomic Level. Advanced Materials.

[25] Chen Y (2016). A Self-Quenching-Resistant Carbon-Dot Powder with Tunable Solid-State Fluorescence and Construction of Dual-Fluorescence Morphologies for White Light-Emission. Advanced Materials.

[26] Xie Z, Wang F, Liu C-Y (2012). Organic–Inorganic Hybrid Functional Carbon Dot Gel Glasses. Advanced Materials.

[27] Qu S (2014). Highly Luminescent Carbon-Nanoparticle-Based Materials: Factors Influencing Photoluminescence Quantum Yield. Particle & Particle Systems Characterization.

[28] Huang JJ (2014). An Easy Approach of Preparing Strongly Luminescent Carbon Dots and Their Polymer Based Composites for Enhancing Solar Cell Efficiency. Carbon.

[29] Zhang L, Wang E (2014). Metal nanoclusters: New fluorescent probes for sensors and bioimaging. Nano Today.

[30] Shang L, Dong S, Nienhaus GU (2011). Ultra-small fluorescent metal nanoclusters: Synthesis and biological applications. Nano Today.

[31] Luo Z (2012). From Aggregation-Induced Emission of Au(I)–Thiolate Complexes to Ultrabright Au(0)@Au(I)–Thiolate Core–Shell Nanoclusters. Journal of the American Chemical Society.

[32] Chen Y-S, Choi H, Kamat PV, Metal-Cluster-Sensitized Solar Cells. A (2013). New Class of Thiolated Gold Sensitizers Delivering Efficiency Greater Than 2%. Journal of the American Chemical Society.

[33] Chen Y-S, Kamat PV (2014). Glutathione-Capped Gold Nanoclusters as Photosensitizers. Visible Light-Induced Hydrogen Generation in Neutral Water. Journal of the American Chemical Society.

[34] Talite MJA (2016). Solid-state, ambient-operation thermally activated delayed fluorescence from flexible, non-toxic gold-nanocluster thin films: towards the development of biocompatible light-emitting devices. Nanotechnology.

[35] Huang HY (2017). Eco-Friendly Luminescent Solar Concentrators with Low Reabsorption Losses and Resistance to Concentration Quenching based on Aqueous-Solution-Processed Thiolate-Gold Nanoclusters. Nanotechnology.

[36] Tian D, Qian Z, Xia Y, Zhu C (2012). Gold Nanocluster-Based Fluorescent Probes for Near-Infrared and Turn-On Sensing of Glutathione in Living Cells. Langmuir.

[37] Mei J, Leung NLC, Kwok RTK, Lam JWY, Tang BZ (2015). Aggregation-Induced Emission: Together We Shine, United We Soar!. Chemical Reviews.

[38] Wu Z, Jin R (2010). On the Ligand’s Role in the Fluorescence of Gold Nanoclusters. Nano Letters.

[39] Goswami N (2016). Luminescent Metal Nanoclusters with Aggregation-Induced Emission. The Journal of Physical Chemistry Letters.

[40] Goswami N, Lin F, Liu Y, Leong DT, Xie J (2016). Highly Luminescent Thiolated Gold Nanoclusters Impregnated in Nanogel. Chemistry of Materials.

[41] Tian R (2015). Localization of Au Nanoclusters on Layered Double Hydroxides Nanosheets: Confinement-Induced Emission Enhancement and Temperature-Responsive Luminescence. Advanced Functional Materials.

[42] Stamplecoskie KG, Chen Y-S, Kamat PV (2014). Excited-State Behavior of Luminescent Glutathione-Protected Gold Clusters. The Journal of Physical Chemistry C.

[43] Chang H-C, Chang Y-F, Fan N-C, Ho J-aA (2014). Facile Preparation of High-Quantum-Yield Gold Nanoclusters: Application to Probing Mercuric Ions and Biothiols. ACS Applied Materials & Interfaces.

[44] Jiang J (2014). Oxidation at the Core–Ligand Interface of Au Lipoic Acid Nanoclusters That Enhances the Near-IR Luminescence. The Journal of Physical Chemistry C.

[45] Pramanik G (2018). Gold nanoclusters with bright near-infrared photoluminescence. Nanoscale.

[46] Wang Z (2017). *In Situ* Fabrication of Flexible, Thermally Stable, Large-Area, Strongly Luminescent Copper Nanocluster/Polymer Composite Films. Chemistry of Materials.

[47] Ai L (2017). Copper inter-nanoclusters distance-modulated chromism of self-assembly induced emission. Nanoscale.

[48] Schlegel, G., Bohnenberger, J., Potapova, I. & Mews, A. *Fluorescence Decay Time of Single Semiconductor Nanocrystals*. Vol. 88 (2002). 10.1103/PhysRevLett.88.137401 11955124

[49] Filatov MA, Baluschev S, Landfester K (2016). Protection of densely populated excited triplet state ensembles against deactivation by molecular oxygen. Chemical Society Reviews.

[50] Uoyama, H., Goushi, K., Shizu, K., Nomura, H. & Adachi, C. Highly efficient organic light-emitting diodes from delayed fluorescence. *Nature* **492**, 234, 10.1038/nature11687, https://www.nature.com/articles/nature11687#supplementary-information (2012). 10.1038/nature11687 23235877

[51] Fernando BD, Thomas JP, Andrew PM (2017). Photophysics of thermally activated delayed fluorescence molecules. Methods and Applications in Fluorescence.

[52] Li Z, Yao W, Kong L, Zhao Y, Li L (2015). General Method for the Synthesis of Ultrastable Core/Shell Quantum Dots by Aluminum Doping. Journal of the American Chemical Society.

[53] Zheng Q (2014). Ultra-stable organic fluorophores for single-molecule research. Chemical Society Reviews.

[54] Lu X (2016). Combination of chemical etching of gold nanoclusters with aggregation-induced emission for preparation of new phosphors for the development of UV-driven phosphor-converted white light-emitting diodes. Journal of Materials Chemistry C.

[55] Bhandari S, Pramanik S, Khandelia R, Chattopadhyay A (2016). Gold Nanocluster and Quantum Dot Complex in Protein for Biofriendly White-Light-Emitting Material. ACS Applied Materials & Interfaces.

[56] Maiti DK, Roy S, Baral A, Banerjee A (2014). A fluorescent gold-cluster containing a new three-component system for white light emission through a cascade of energy transfer. Journal of Materials Chemistry C.

[57] Li D, Chen Z, Mei X (2017). Fluorescence enhancement for noble metal nanoclusters. Advances in Colloid and Interface Science.

[58] Julia G (2018). Stable White Light-Emitting Biocomposite Films. Advanced Functional Materials.

[59] Sugiuchi M (2017). Aggregation-Induced Fluorescence-to-Phosphorescence Switching of Molecular Gold Clusters. Journal of the American Chemical Society.

[60] Wang F, Zhang X, Zhang Z, He C (2011). *In situ* formation and ordered assembly of gold nanoclusters to nano-ribbons at the oil/water interface. Journal of Materials Chemistry.

[61] Zhang J (2015). Microwave-assisted synthesis of photoluminescent glutathione-capped Au/Ag nanoclusters: A unique sensor-on-a-nanoparticle for metal ions, anions, and small molecules. *Nano*. Research.

