# Supplementary material for: Coordination-induced emission enhancement in gold-nanoclusters with solid-state quantum yields up to 40% for eco-friendly, low-reabsorption nano-phosphors
Source: Sci Rep. 2019 Mar 11;9:4053. doi: 10.1038/s41598-019-40706-3 (PMC6411768; doi:10.1038/s41598-019-40706-3)
Supplement: Supplementary file 1 — Supporting Information [file 41598_2019_40706_MOESM1_ESM.docx]

**Supporting Information**

**Coordination-induced emission enhancement in gold-nanoclusters with solid-state quantum yields up to 40% for eco-friendly, low-reabsorption nano-phosphors**

Hsiu-Ying Huang ^1#^, Kun-Bin Cai ^1,2#^, Maria Jessabel Talite^3^, Wu-Ching Chou^3^, Po-Wen Chen ^2^*, Chi-Tsu Yuan^1,4^*

^1^Department of Physics, Chung Yuan Christian University, Taoyuan, Taiwan

^2^Physics Division, Institute of Nuclear Energy Research, Taoyuan, Taiwan

^3^Department of ElectroPhysics, National Chiao Tung University, Hsin Chuu, Taiwan

^4^R&D Center for Membrane Technology, Chung Yuan Christian University, Taoyuan 320, Taiwan

**Fig. S1: Optical properties of solid GSH-AuNCs powders.**

Optical absorption and PL emission spectra for the aggregated GSH-AuNCs powders were shown below. Compared with GSH-AuNCs dispersed in an aqueous solution, the PL-QYs of solid GSH-AuNC powders in the aggregated state can be enhanced (from ~1% to ~8%) accompanied with a spectral blue shift, thus exhibiting conventional aggregation-induced emission enhancement.

**Fig. S2: Analyses of time-resolved PL decay profiles.**

The time-resolved PL decay profiles for GSH-AuNCs dispersed in an aqueous solution, solid GSH-AuNCs aggregated powders, and Zn-GSH-AuNC powders are shown below. Those curves can be fitted using a stretched exponential function with the deconvolution of instrument response function, $I(t)=I_{0}\exp(-\frac{t}{\tau})^{\beta}+I_{b}$, where $I_{0},\beta,\tau,I_{b}$ represent the PL intensity at zero time delay, stretching parameter, characteristic time, and background intensity, respectively. After obtaining $\beta,\tau$ from the experimental data, the average PL lifetime can be deduced using this equation, $\left\langle\tau\right\rangle=\frac{\tau}{\beta}\Gamma(\frac{1}{\beta})$, where $\Gamma$is the gamma function. The average PL lifetimes were calculated to be $\text{\textasciitilde1.2}\mu s$, $\text{\textasciitilde4.4}\mu s$, and$\text{\textasciitilde10.3}\mu s$ for GSH-AuNCs dispersed in an aqueous solution, aggregated GSH-AuNC powders, and assembled Zn-GSH-AuNCs, respectively.

**
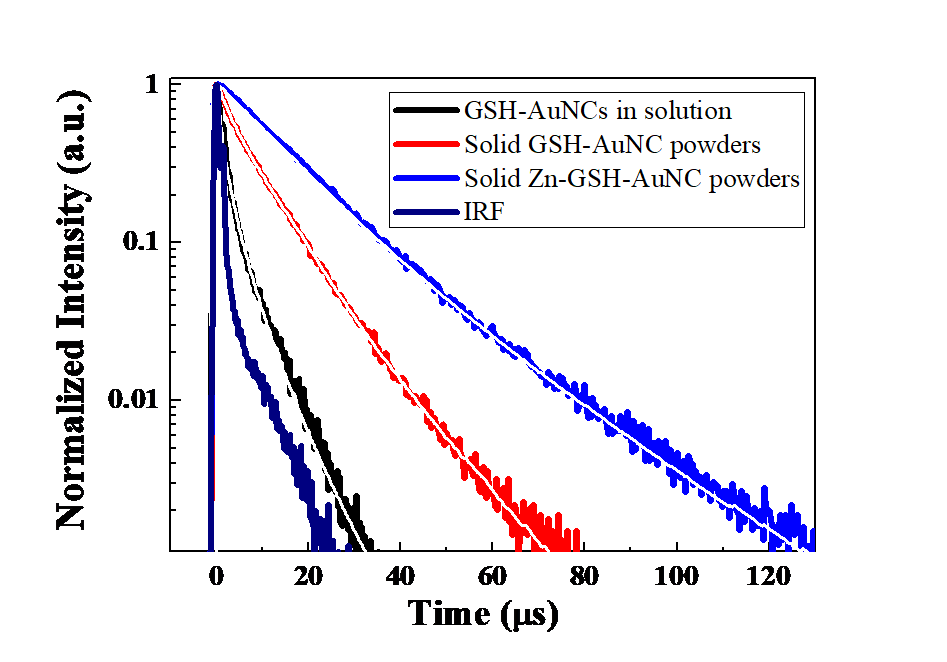
**

**Fig. S3: Forster radius calculation and FRET efficiency.**

The FRET efficiency can be determined by this equation, $E_{FRET}=\frac{1}{1+(d/R_{0})^{6}}$, where $d,R_{0}$ are the separation distance and Forster radius. The Forster radius can be calculated using this equation, $R_{0}=0.211[\kappa^{2}n^{-4}\varphi_{D}J(\lambda)]^{1/6}$where $\kappa\text{,}n,\varphi_{D},J(\lambda)$represent orientation factor, refractive index, emission quantum yields and spectral overlap integral, respectively. The spectral overlap integral can be defined as, $J(\lambda)=\int_{0}^{\infty} F_{D}(\lambda)\varepsilon_{A}(\lambda)\lambda^{4}d\lambda$, where $F_{D}(\lambda),\varepsilon(A)$is the normalized PL spectrum and molar extinction coefficient. According to our experimental result, the spectral overlap integral is $J\sim3.3\times10^{11}M^{-1}cm^{-1}nm^{4}$, and the resulting Forster radius is 0.9~1.0 nm. From the high-resolution TEM image, the separation distance between Zn-GSH-AuNCs is at least ~3 nm, leading to negligible FRET efficiency, as shown below.


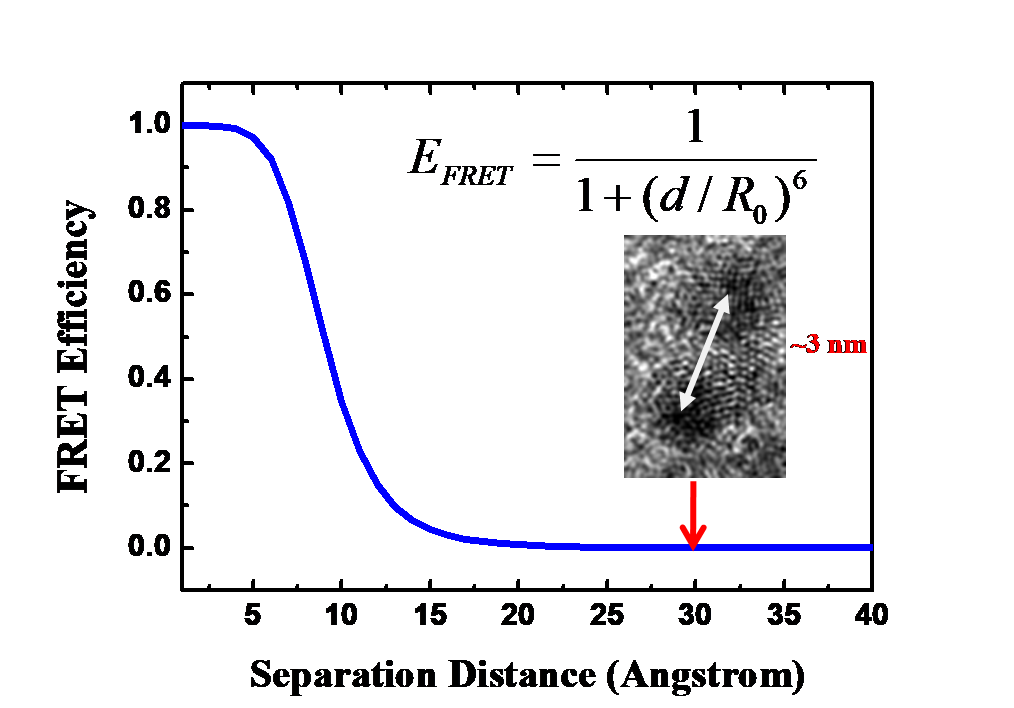


**Fig. S4：The photograph of GSH-AuNCs add different metal ions and GSH-CuNCs add Zn2+ ion under UV light.**

Apart from Zn^2+^ additives presented in this manuscript, we have also tested Al^3+^ and Cu^2+^ cations as the additives to see the effects on the PL intensity of GSH-AuNCs. As shown below, the addition of Al^3+^ cation has a similar effect as Zn^2+^ to cause PL enhancement, while the Cu^2+^ cations conversely reduce the PL intensity. In addition, we also applied the same method to thiolated copper nanoclusters (GSH-CuNCs), as also shown below. As the case of GSH-AuNCs, similar PL enhancement was clearly observed for GSH-CuNCs upon the addition of Zn^2+^.

**
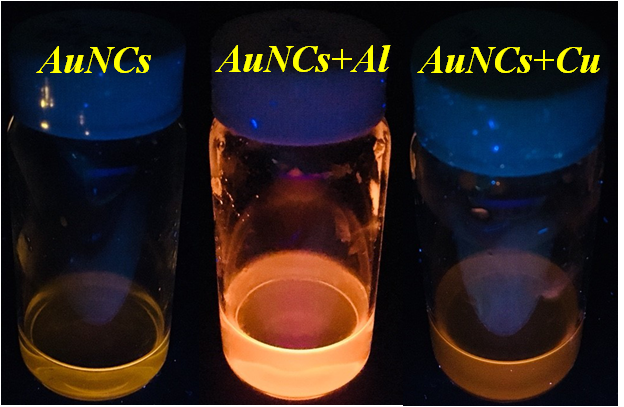

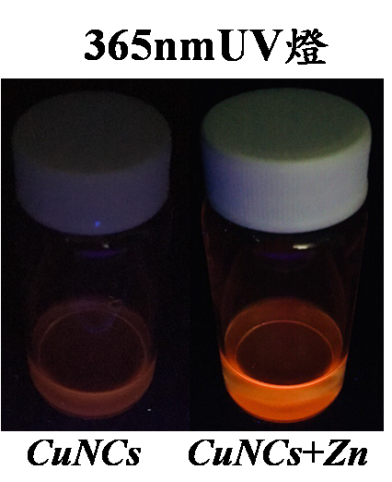
**
